# Hepatic melanomacrophage centers in the arctic cultured fish *Cyclopterus lumpus* are not indicative of its health state

**DOI:** 10.1016/j.aquaculture.2023.740417

**Published:** 2024-02-25

**Authors:** L. Passantino, A. Corriero, C. Pousis, R. Zupa, A. Perillo, J. Superio, J. Kumari Swain, A. Foss, J. Galindo-Villegas, G. Ventriglia

**Affiliations:** a DiMePRe-J, University of Bari Aldo Moro, Valenzano 70010, Italy; b Department of Veterinary Medicine, University of Bari Aldo Moro, Valenzano 70010, Italy; c UiT The Artic University of Norway, N-9037 Tromsø, Norway; d Akvaplan-niva, Fram Centre, 9296 Tromsø, Norway; e Department of Genomics, Faculty of Biosciences and Aquaculture, Nord University, Bodø, Norway

**Keywords:** CYP1A, Health markers, immunity, Necrosis, Non-model vertebrate

## Abstract

The lumpfish, *Cyclopterus lumpus*, holds significant promise as a candidate for large-scale aquaculture production, particularly in its role as a cleaner fish used to manage sea lice infestations in Atlantic salmon *Salmo salar* farming. Melanomacrophage centers (MMCs) represent polymorphic structures present in the hemolymphopoietic organs of various vertebrates, serving as a widely applicable histological indicator of the fish immune and health status. This study aims to investigate the histochemical characteristics of MMCs within lumpfish livers and to compare MMC density between hatchery-produced (farmed) and wild individuals. Liver samples were collected from 34 lumpfish and subjected to a range of staining techniques, including haematoxylin-eosin, Azan-Mallory's trichrome, Masson-Fontana, Perls-Van Geison, Mallory's hemofuscin, immunohistochemical detection of cytochrome P450 monooxygenase 1 A (CYP1A), and the terminal deoxynucleotidyl transferase-mediated d'UTP nick-end labelling (TUNEL) method. Hepatocytes from hatchery-produced males exhibited notably high lipid content. Additionally, cells showing positive staining with Masson-Fontana, likely associated with the monocyte/macrophage lineage, were identified. Furthermore, small MMCs containing melanin, lipofuscin-ceroids, and ferric ions were detected. While the density of single monocytes/macrophages was markedly higher in hatchery-produced males, no significant discrepancies in MMCs density were observed between wild and hatchery-produced fish, or between males and females of the same origin. The study also revealed the presence of necrotic foci, characterized by hypertrophic hepatocytes positive for both TUNEL and CYP1A staining. These hypertrophic hepatocytes displayed large lipid droplets and pycnotic nuclei, with hatchery-produced males showing a higher numerical density of such foci. In contrast to findings in other fish species, the study found that MMCs did not appear to serve as reliable markers of health status in lumpfish. This conclusion was reached as MMCs density did not exhibit a correlation with necrotic foci or hepatocyte lipid content.

## Highlights

• A comparison of hepatic micrographs was conducted between farmed lumpfish and their wild counterparts.
• The histological structure of hepatic lumpfish melanomacrophage centers (MMC) was chosen as an indicator of health status
• Numerical density of hepatic macrophages was higher in farmed fish, while MMCs were unaffected across conditions and sexes.
• In lumpfish, hepatic MMCs showed no correlation with necrotic foci or hepatocyte lipid content.
• Hepatic MMCs in lumpfish do not serve as reliable markers of immune or health status.

## 1 Introduction

The lumpfish *Cyclopterus lumpus* (L. 1758) is a semi-pelagic fish with a wide distribution in the arctic and sub-arctic regions of both the eastern and western North Atlantic Ocean (Imsland et al., 2019). During its life cycle, it inhabits coastal areas during the juvenile phase (Moring, 2001), venture into open sea foraging grounds, and eventually returns to coastal regions for spawning (Eriksen et al., 2014; Holst, 1993). Notably, the lumpfish has garnered commercial interest due to its mature oocytes being used to produce a caviar substitute (Kasper et al., 2014) and its recent adoption as a cleaner fish in the removal of sea lice from Atlantic salmon *Salmo salar* (Myhre Jensen et al., 2020). Consequently, the demand for lumpfish has led to a rapid increase in aquaculture production, with over 30 million individuals being produced in 2017, compared to around 400,000 in 2012 (Norwegian Directorate of Fisheries (NDF), https://www.fiskeridir.no/Akvakultur).

At present, significant research and industrial effort are directed towards supplying salmon farms with farmed lumpfish (Andersson et al., 2023; Imsland et al., 2015). Despite the involvement of approximately 25–30 fish farms in lumpfish production, most eggs are sourced from two farms, primarily obtained from wild-caught fish (NDF, online). This highlights the importance of transitioning from capture-based farming to a self-sustained aquaculture model, which not only alleviates fishery pressure on the species, classified as “near threatened” in its north-eastern population by the International Union for Conservation of Nature (https://www.iucnredlist.org), but also meets the needs of the salmon farming industry. This shift would yield significant benefits for the environment, salmon well-being, and consumer health. The transition necessitates the development of species-specific techniques for reproduction control, larval rearing, and growth. Amidst this, a key challenge for the modern fish farming industry is to minimize stress, optimize fish welfare and immune status, comply with ethical standards, provide high-quality fish food to consumers, and enhance the economic sustainability of aquaculture operations (Toni et al., 2019; Montalban-Arques et al., 2015; Meling et al., 2022).

The escalating demand for live lumpfish within the salmon industry has catalyzed significant efforts towards establishing a reliable, large-scale production technology for this species in captivity (Andersson et al., 2023). Given the relatively recent rise of industrial interest in lumpfish, our understanding of its biology remains limited. Currently, our knowledge primary centers around crucial aspects involving wild and hatchery lumpfish reproduction (Andersson et al., 2023; Imsland et al., 2018; Kennedy, 2018; Lein et al., 2023), sex determination (Chaiyasut et al., 2023), feeding preferences (Imsland et al., 2018), health and prophylaxis (Dang et al., 2021) growth under farming conditions (Imsland et al., 2014), effects of dietary lipids (Berge et al., 2023), and the consequences of substituting fishmeal with plant protein on digestive physiology (Willora et al., 2022).

The liver, a pivotal organ, performs a multitude of essential functions encompassing both anabolic and catabolic processes, as well as the metabolism of xenobiotics, rendering it a robust indicator of health status of fish (Ardeshir et al., 2022). Over the past five decades, the presence of polymorphic structures resembling granulomas, known as melanomacrophage centers (MMCs), within hemolymphopoietic organs including the liver, has captured the attention of fish biologists and pathologists (Steinel and Bolnick, 2017; Stosik et al., 2019). These MMCs, contain pigments like melanin, hemosiderin, and lipofuscin, resulting from the aggregation of macrophages and lymphocytes, phagocytosed cell fragments, and residues of reticulo-endothelial cell activity (Agius and Roberts, 2003; Barst et al., 2015; Fishelson, 2006; Koppang et al., 2005). As a result, numerous analyses employing various techniques, ranging from classical histology to transcriptomics, have indicated that MMCs play a key role in the general immune response of fish. *In vitro* and *in vivo* research has predominantly focused on exploring the innate and adaptive humoral and cell-mediated immune mechanisms, as well as crucial pathways involved in anti-bacterial and anti-viral defenses in lumpfish (Eggestøl et al., 2018; Gnanagobal et al., 2021; Rao et al., 2023; Rønneseth et al., 2015). Furthermore, attempts have been made to establish a foundational framework for effective immunization protocols against major pathogens that could impact farmed lumpfish (Dang et al., 2021; Erkinharju et al., 2019). However, despite the accumulating knowledge, insights into the immune system of lumpfish remain relatively limited.

Hence, the primary objective of the present study was to delve into the histochemical attributes of MMCs within lumpfish livers and to undertake a comparative analysis of the overall MMC characteristics between hatchery-produced and wild lumpfish of both sexes. The overarching goal was to enhance our comprehension of the functional aspects of MMCs in lumpfish, offer insights into their immunological implications, and evaluate their potential suitability as indicators of the health status within this species.

## 2 Materials and methods

### 2.1 Ethical statement

For this study, both wild and farmed lumpfish were utilized. Wild fish were captured commercially. Farmed fish were bred from eggs obtained from Akvaplan-Niva (Tromsø, Norway) and raised under standard farming condition. The use of the farmed fish in this study was approved by the Norwegian Animal Research Authority, FDU (Mattilsynet ID-29566). All procedures involving animals were conducted following the guidelines for the treatment of animals in behavioral research and teaching (Simmonds, 2018), and the “Directive 2010/63/EU of the European parliament on the protection of animals used for scientific purposes” (Olsson et al., 2016). The authors adhered to the ARRIVE guidelines.

### 2.2 Sample collection

Liver samples measuring approximately 0.5 × 0.5 × 0.5 cm were collected from a total of 34 apparently healthy lumpfish individuals, including 17 wild fish (eight females and nine males) and 17 naïve hatchery-produced (nine females and eight males) (Table 1). The hatchery-produced fish were generated from eggs obtained from wild-caught lumpfish, which were kept in 25 m^3^ tanks at the Akvaplan-niva facility in Tromsø, Norvay. following standard farming practices. After collection, the liver samples were fixed in Bouin's solution for further analysis. Additionally, the hepato-somatic index (HIS) was calculated using the formula 100xLiver Mass/Body Mass.

**Table 1:** Origin, sex, body mass and hepato-somatic index (HIS) recorded for the sampled lumpfish.

**Fish origin**	**Sex**	**Fish (n)**	**Body mass (kg)**	**HSI**
Wild	Females	8	3.6 ± 1.1^a^	2.2 ± 0.7^a^
	Males	9	1.7 ± 0.3^b^	2.5 ± 0.8^a^
Hatchery-produced	Females	9	2.6 ± 1.3^c^	3.5 ± 1.1^b^
	Males	8	0.8 ± 0.3^d^	2.2 ± 0.8^a^

Different letters indicate statistically significant differences (ANOVA; *P* > 0.05).

### 2.3 Histological, immunohistochemical and TUNEL staining

Liver samples were prepared for histological analysis by embedding them in paraffin wax, following a sequence of steps that included dehydration using escalating concentrations of ethanol and clarification in xylene. From each sample, five to ten liver sections, each measuring five μm thickness, were sectioned and subjected to various staining methods aimed at visualizing different components.

For general histological examination, haematoxylin-eosin and Azan-Mallory's trichrome (Merk, Darmstadt, Germany) stainings were used. Masson-Fontana (Bio-Optica, Milan, Italy), Perls-Van Gieson (Bio-Optica, Milan, Italy) and Mallory's hemofuscin stainings were utilized to detect melanin, ferric ions of hemosiderin and lipofuscin–ceroids, respectively. Alongside these staining techniques, immunohistochemical detection of cytochrome P450 monooxygenase 1 A (CYP1A), a recognized immune mediator and biomarker of fish exposure to aquatic pollutants, was conducted on liver sections of all the sampled fish. The procedure followed to a previously described method (Basilone et al., 2018). The sections were deparaffinization and rehydrated, and non-specific binding sites were blocked with normal horse serum (NHS). The sections were then incubated with a polyclonal anti-fish CYP1A peptide antibody (Biosense Laboratories, Bergen, Norway) diluted 1:500 in PBS containing 0.1% bovine serum albumin (BSA); the visualization was achieved using the Vectastain Universal Elite Kit (Vector, Burlingame, CA). Nuclear counterstaining was obtained by a quick section treatment (20 s) with a ready-to use solution of haematoxylin (Vector, Burlingame, CA). Furthermore, the terminal deoxynucleotidyl transferase-mediated d'UTP nick-end labelling (TUNEL) method (*In Situ Cell Death Detection Kit, AP*) (Roche Diagnostics) was used on deparaffinized sections to label fragmented DNA strands of dead cells. The sections were treated with a permeabilization solution of 0.1% Triton X-100 in 0.1% sodium citrate and then incubated with the reaction mixture. Terminal deoxynucleotidyl transferase was diluted 1:20 in TUNEL Dilution Buffer (Roche Diagnostics), and a ready-to-use solution of nitro blue tetrazolium chloride/5-bromo-4-chloro-3-indolyl phosphate, toluidine salt (NBT/BCIP) (Roche Diagnostics) was used as a substrate for the signal conversion. According to the manufacturer's instruction manual, the TUNEL method is designed to label apoptotic cells, but it stains also advanced necrotic cells.

### 2.4 Relative quantification of necrotic foci and MMCs

The density of single monocytes/macrophages (number of monocytes/macrophages/mm^2^ hepatic parenchyma), the density of melanomacrophage centers (μm^2^ MMCs/mm^2^ hepatic parenchyma), and the number of necrotic foci present per square millimeter of hepatic parenchyma, were measured on liver sections stained with haematoxylin-eosin and photographed with a digital camera (DFC 420, Leica) connected to a light microscope (DIAPLAN, Leitz). Measurements were taken using an image analysis software (Leica Application Suite, version 3.3.0).

### 2.5 Statistical analysis

Data normal distribution was assessed by Shapiro-Wilk W test. Differences in body mass, HIS, density of monocytes/macrophages as well as in the areal density of melanomacrophage centers were assessed by an ANOVA followed by Duncan's new multiple range *post hoc* test. The number of necrotic foci did not show normal distribution and differences were estimated by means of a Kruskal-Wallis ANOVA test for non-parametric comparisons of multiple independent groups; then, Mann-Whitney *U* test was used to test the differences between all sample pairs. All the results are presented as means ± SD, and the statistical probability significance was established at the *P* < 0.05 level.

## 3 Results

**Fig. 1:**
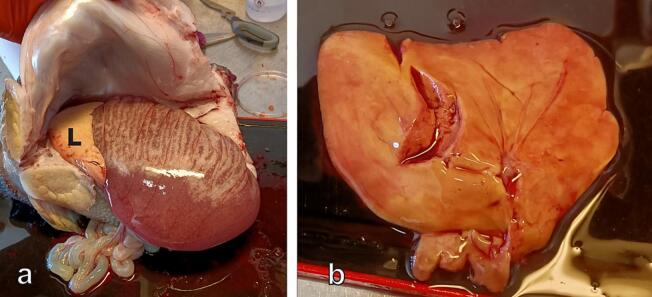
(**a**) Illustration of the peritoneal cavity in a female lumpfish, where a pale-yellow liver (L) covers the cranial portion of well-developed ovaries that have undergone partial spawning. (**b**) Dissected liver from a female lumpfish, captured after sampling for analysis, displaying an orange color. (For interpretation of the references to color in this figure legend, the reader is referred to the web version of this article.)

The lumpfish liver displays a multilobulated structure with indented margins and variable color, ranging from pale yellow to bright orange (Fig. 1a, b).

**Fig. 2:**
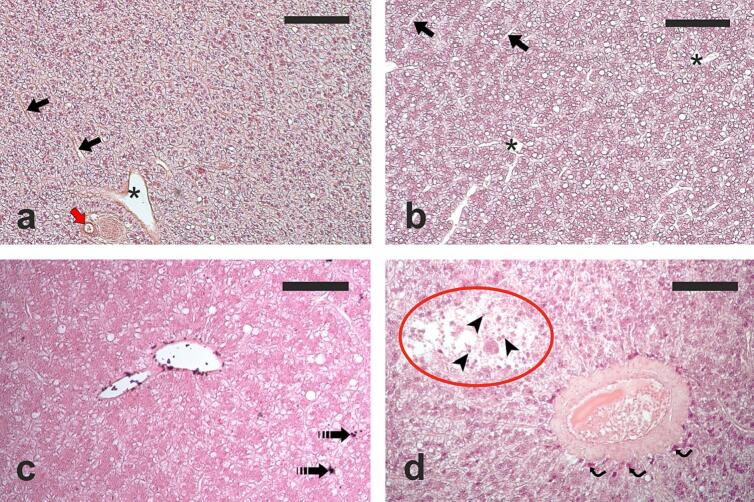
Micrographs of hepatic sections: (**a**) from a wild female lumpfish, and (**b**) a hatchery-produced male lumpfish. Notably the liver of the hatchery-produced male exhibits a higher density of optically empty vacuoles, indicative of lipid droplets. (**c**) Micrograph showing hepatic section of a hatchery-produced male, highlighting endovasal and extravasated cells belonging to the monocyte/macrophage lineage (white venules), along with small melanomacrophage centers (dashed arrows). (**d**) Micrograph depicting hepatic section of a wild female lumpfish, revealing a necrotic focus (encircled area) characterized by hypertrophic hepatocytes with pycnotic nuclei (arrowheads), accompanied by pronounced inflammatory leukocyte infiltration (curved arrows) surrounding a blood vessel. Haematoxylin-eosin staining applied in (**a**), (**b**), and (**d**), while Masson-Fontana staining indicates melanin presence in (**c**). Scale bars = 200 μm in (a) and (**b**), and 100 μm in (**c**) and (**d**). Asterisk shows a branch of the portal vein; black arrow denotes a sinusoid; red arrow points to a branch of the hepatic artery. (For interpretation of the references to color in this figure legend, the reader is referred to the web version of this article.)

The liver exhibits polygonal hepatocytes with a moderately basophilic cytoplasm containing variable-sized lipid droplets and a central nucleus, arranged in branched and anastomosed double-cell cords, surrounded by sinusoids (Fig. 2). Hatchery-produced fish showed a higher lipid accumulation in the liver (Fig. 2a), compared to wild fish (Fig. 2b), with males having a higher lipid content than females of the same origin.

However, the hepatosomatic index of hatchery-produced females was significantly higher (*P* < 0.05) than wild females and males of both origins (Table 1).

Positive cells to the Masson-Fontana staining for melanin, likely belonging to the monocyte/macrophage lineage, were frequently observed in the liver sections (Fig. 2c). These cells were found in the *tunica intima* and *adventitia* of portal vein branches as well as in the tunica *adventitia* of artery vessels (Fig. 2c, d).

**Fig. 3:**
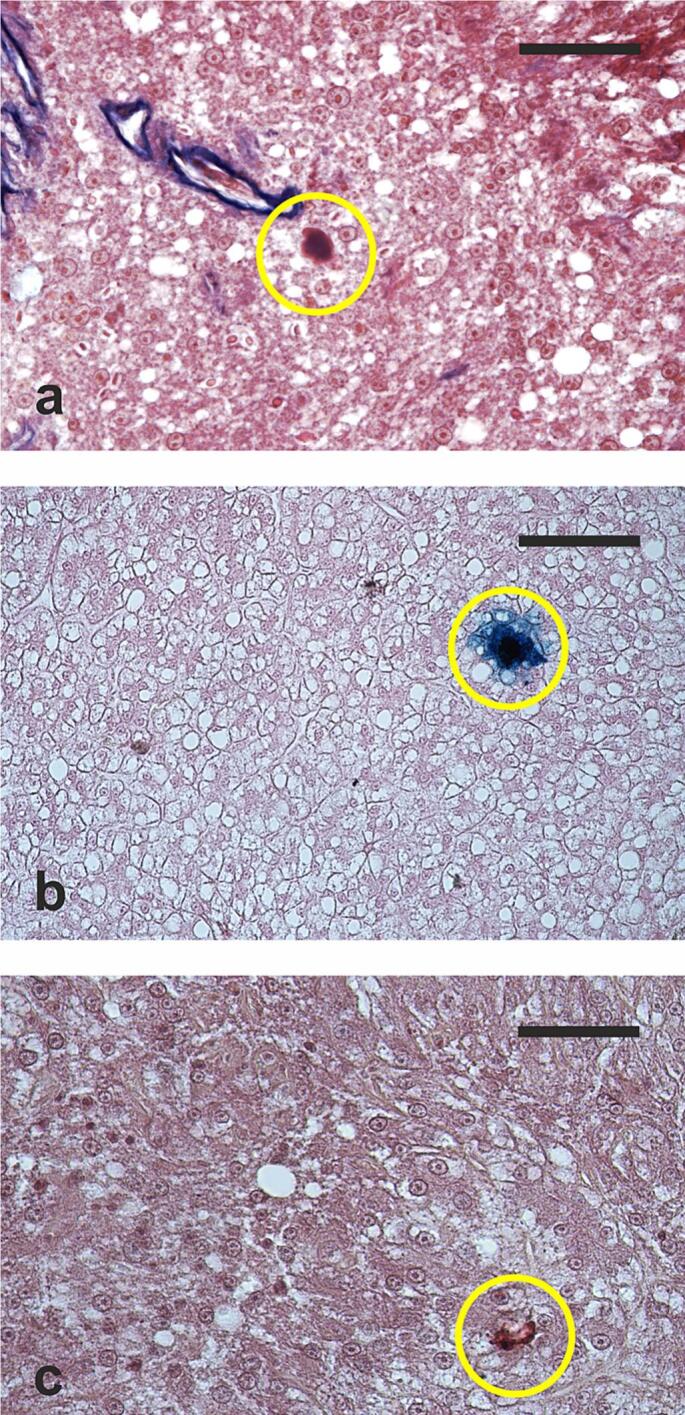
(**a**) Micrograph of a hepatic section from a hatchery-produced male lumpfish, showcasing a small melanomacrophage center located proximal to a blood vessel. Azan-Mallory's trichrome staining. (**b**) Hepatic section micrograph of a hatchery-produced male, displaying a melanomacrophage center containing ferric ions positive stained through Perl's-van Gieson technique. (**c**) Micrograph illustrating hepatic section from a wild male, portraying a small melanomacrophage center containing material positively stained with Mallory's hemofuscin for liposfuscin-ceroids. Encircled areas delineate a small melanomacrophage center. Scale bars = 50 μm in (**a**) and (**c**), 100 μm in (**b**).

Small MMCs, composed of aggregates of cells and pigments, were also present and they contained melanin, ferric ions, and lipofuscin-ceroids (Fig. 3).

**Fig. 4:**
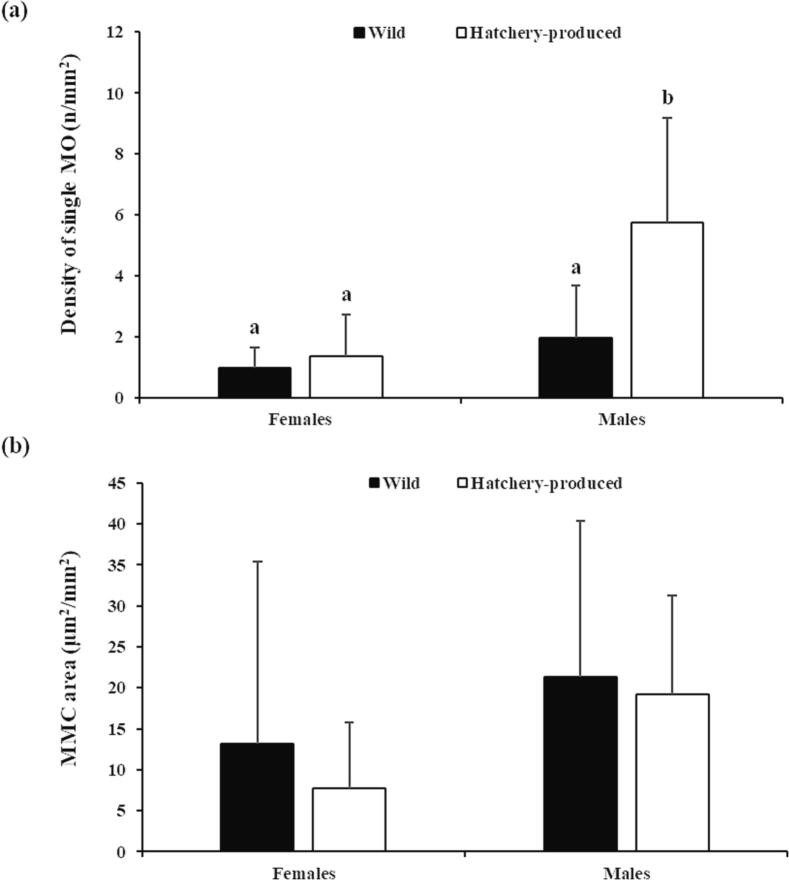
Comparison of numerical density of cells pertaining to the monocyte/macrophage lineage (positively stained for melanin using Masson-Fontana staining) (a), and areal density of melanomacrophage centers (b) in the liver of both wild (*n* = 8♀/♂) and hatchery-produced (*n* = 9/8 ♀/♂) lumpfish. Different letters indicate statistically significant differences (Mean ± SD; ANOVA; P < 0,05).

The quantitative analysis revealed a significant higher density of single monocytes/macrophages in hatchery-produced males compared to other groups (*P* < 0.05) (Fig. 4a). Although the subjective histological evaluation suggested the presence of a higher amount of MMCs in males, likely due to a high individual variability, no statistically significant differences were found in the density of these structures among groups (*P* = 0.384) (Fig. 4b).

**Fig. 5:**
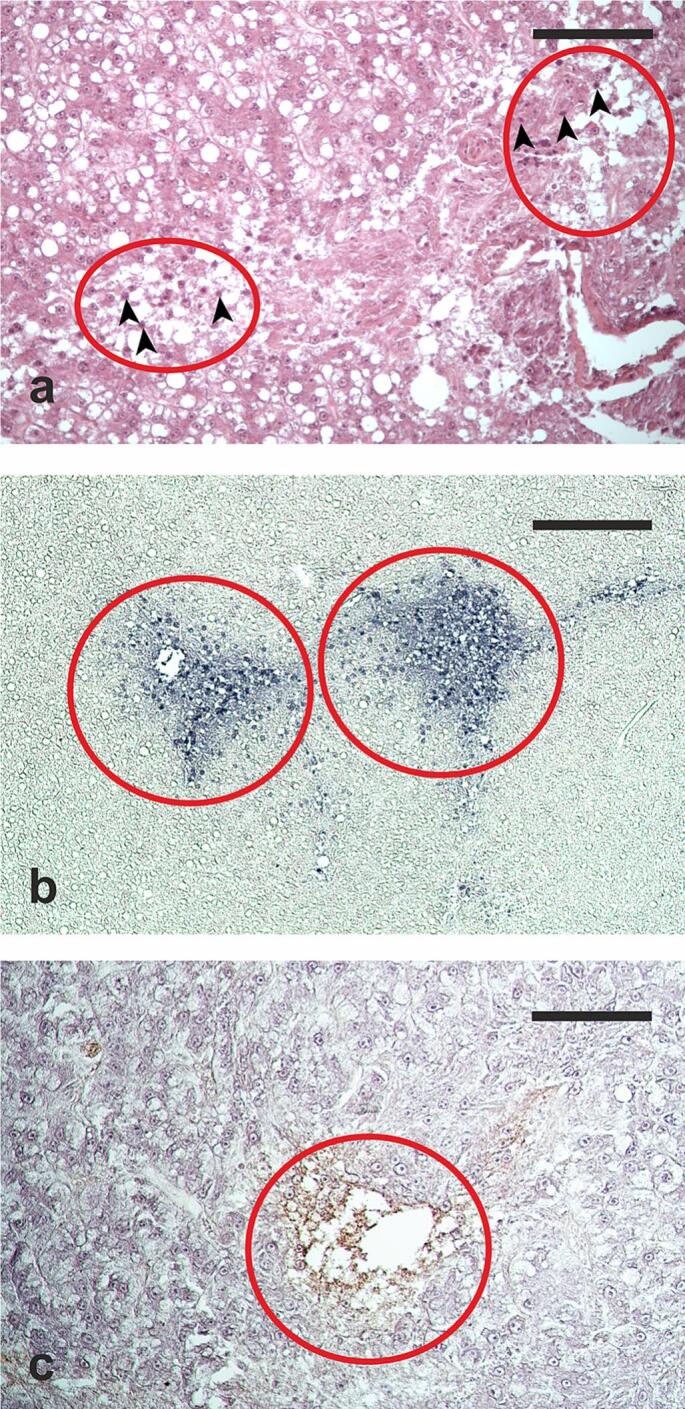
Hepatic section micrograph of a hatchery-produced male, (**a**) highlighting two necrotic foci. Haematoxylin and eosin staining applied; (**b**) necrotic foci containing numerous TUNEL-positive hepatocytes (displayed as blue-stained cells); (**c**) immunostained with anti-CYP1A antibodies, revealing some necrotic hepatocytes exhibiting robust cytochrome 450 monooxygenase immunopositivity. Arrowheads indicate pycnotic nuclei within hepatocytes. Encircled areas delimit necrotic foci. Scale bars = 100 μm in (**a**) and (**c**), 200 μm in (**b**). (For interpretation of the references to color in this figure legend, the reader is referred to the web version of this article.)

Parenchymal areas with lower cell density, characterized by hypertrophic hepatocytes containing large lipid droplets and a pycnotic nucleus (Fig. 5a), were observed in all analyzed samples. Numerous TUNEL-positive hepatocytes were observed in these areas, that were identified as necrotic foci (Fig. 5b). A faint anti-CYP1A immunostaining was observed in the liver sections, except for some necrotic hepatocytes showing a more intense staining (Fig. 5c).

**Fig. 6:**
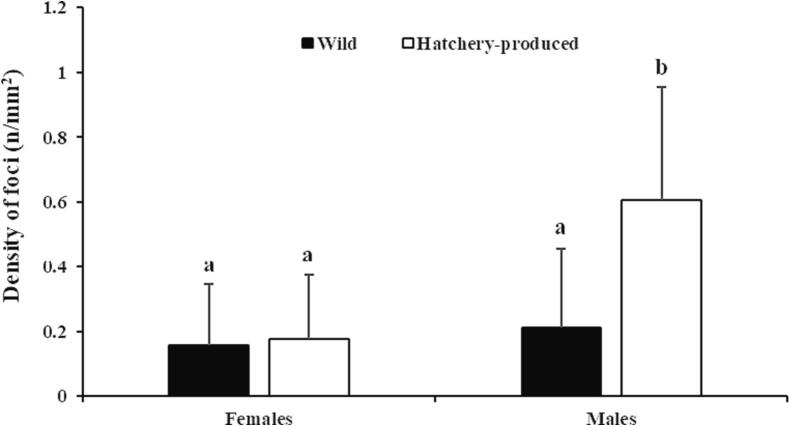
Comparative illustration of necrotic foci density in the liver of wild (*n* = 8/9 ♀/♂) and hatchery-produced (*n* = 9/8 ♀/♂) lumpfish. Different letters indicate statistically significant differences between groups (Mean ± SD; Mann-Whitney *U* test; P < 0.05).

Statistically significant differences in the numerical density of necrotic foci were not found between wild and hatchery-produced females (*P* = 0.552), but hatchery-produced males had a higher number of necrotic foci compared to the other groups (*P* < 0.05) (Fig. 6).

## 4 Discussion

In this research, we shed light on the histological attributes of MMCs in lumpfish livers and provides insights into their density variations across different fish populations. The findings from this study contribute to our understanding of lumpfish health indicators and their potential implications for aquaculture practices.

Our findings revealed that the liver of lumpfish exhibits a typical architecture commonly observed in teleost fish. This architecture is characterized by a parenchymal arrangement reminiscent of the tubular form previously described by (Akiyoshi and Inoue, 2004). At the cellular level, hepatocytes are organized in a dual layer amidst sinusoids. Interestingly, the liver's coloration ranges from light yellow to bright orange, regardless of origin or sex. Remarkably, this spectrum of coloration does not appear to correlate with the pigmentation observed in the skin and plasma, both of which can also exhibit variations in color and intensity (Staven et al., 2021). Furthermore, our study discerns that this coloration is not directly associated with changes in the analyzed MMCs or the overall health status of the lumpfish. These findings align with the investigations of Eliasen and colleagues, who documented the existence of up to six distinct liver colors in cultured lumpfish. They attributed these variations to differences in lipid class distribution, with the lowest triacyl glyceride values corresponding to light colors (Eliasen et al., 2020). Additionally, they also speculated about potential variations in nutritional status or compromised immune systems as possible contributing factors. However, neither their study nor ours yielded definitive insights into the precise origin of this color variation.

Melanomacrophage centers are commonly prevalent, primarily in the fish head kidney and spleen, but they can also be found in the liver. In the present study, we identified a modest presence of scattered MMCs in the lumpfish liver. Histochemical staining revealed that these MMCs contained: melanin, ferric iron in the form of hemosiderin, a residue from the catabolism of various compounds, and lipofuscin/ceroid pigments resulting from the oxidative polymerization of polyunsaturated fatty acids. These pigments usually accumulate concurrently with various pathological conditions. Previous reports analyzing MMCs in rainbow trout (*Oncorhynchus mykiss*) have hypothesized a link to diverse stressor exposures (Passantino et al., 2002a, Passantino et al., 2002b, Passantino et al., 2004). Moreover, in juvenile Atlantic bluefin tuna (*Thunnus thynnus*) reared in the North Adriatic Sea, heightened liver MMC density corresponded to increased levels of hepatocyte apoptosis and expression of tumor necrosis factor (Corriero et al., 2013; Passantino et al., 2014a). These results suggest that the MMCs play a crucial role in the degradation of both endogenous and exogenous materials, including dead cells and cellular debris, as reported by (Galindo-Villegas and Hosokawa, 2004). Indeed, MMCs act as reservoirs for indigestible catabolic residues arising from various compounds, including hemoglobin, thus functioning as metabolic repositories (Sayed et al., 2022). A clear example emerges from European anchovies (*Engraulis encrasicolus*) captured in marine regions impacted by industrial and agricultural sewages, where an elevated density of MMCs in the liver has been documented (Basilone et al., 2018). Therefore, these observations suggest a direct correlation between fish exposure to persistent organic pollutants and heavy metals and the increase on MMCs number and phenotype. However, in our study, such correlations were not clearly documented.

To further complicate their characterization, it is also known that the size, morphology, and pigment distribution of MMCs vary with age (Matsche et al., 2023), dietary manipulations (Phromkunthong et al., 2015), exposure to infectious agents (Yunis-Aguinaga et al., 2015), the effects of vaccines and adjuvants (Galindo-Villegas et al., 2019), and captivity-induced stress (Nowak et al., 2021). Moreover, at the molecular level, increased MMC density has been linked to the presence of a group of heme-thiolate monoxygeneases derived from cytochrome P450 enzymes, such as CYP1A (Basilone et al., 2018; Passantino et al., 2014b), as well as hepatocyte apoptosis (Corriero et al., 2013). In our study, we observed high individual variability in the areal density of liver MMCs, with statistically significant differences neither between lumpfish of different origin (wild *vs* hatchery-produced) nor between the two sexes. The areal density of MMCs in lumpfish liver was between 5 and 35 μm^2^/mm^2^ that is much lower than that of large, long-lived species in temperate waters, such as the Atlantic bluefin tuna (*Thunnus thynnus*) where 10,000 μm^2^/mm^2^ has been reported (Passantino et al., 2014a), or the greater amberjack (*Seriola dumerili*) with 500–5000 μm^2^/mm^2^ (Passantino et al., 2020). The density was similar to that reported for the small pelagic, short-lived European anchovy, >25 μm^2^/mm^2^ (Basilone et al., 2018) inhabiting cold and warm waters. In this context, the lumpfish musculature is less prominent compared to most commercial fish species, resulting in a lower hematocrit (mean hematocrit value calculate on 16 fish = 18.8%; unpublished data), particularly in comparison with warm water fish. For instance, the European seabass (*Dicentrarchus labrax*) and gilthead sea bream (*Sparus aurata*) exhibit hematocrit values ranging from 20% to 35% (Pascoli et al., 2011; Tort et al., 1998). This physiological variation in blood composition is consistent with the higher oxygen solubility in cold waters, leading to a reduced requirement for erythrocytes as oxygen transporters (Pascoli et al., 2011). In line with this, earlier investigations involving the use of phenylhydrazine as a hemolytic agent or the induction of starvation in goldfish (*Carassius auratus*) led to a rapid and substantial anemia, attributed to an increased metabolic activity between MMCs and iron metabolism (Zapata, 2022). Based on these observations, we propose a hypothesis that the observed low density of MMCs in lumpfish may be linked to a reduced rate of erythrocatheresis, thereby potentially diminishing the activity of macrophages in engulfing apoptotic or necrotic erythrocytes. This in turn, could influence on the number and size of hepatic MMCs available for metabolic recycling structures.

In the present study, we have documented the precence of necrotic foci within the livers of both wild and hatchery-produced fish. These foci were characterized by the hypertrophic hepatocytes containing high lipid content and nuclei displaying signs of condensed chromatin, forming pycnotic structures. The necrotic nature of these deteriorating liver regions was confirmed by the substantial number of TUNEL-positive cells. While the TUNEL method is primarily designed to label apoptotic cells, the manufacturer's manual indicates its applicability to label cells undergoing advanced necrosis as well. Notably, the identification of these necrotic foci was an unexpected revelation, given that no macroscopic abnormalities were noted during the sampling process. As a result, no immediate plans for further investigations were formulated to delve into the origin of these findings. The presence of these necrotic foci presents an ambiguous interpretation. However, the overall minimal immunopositivity of liver sections to CYP1A antibodies contradicts the assumption of exposure to toxic compounds. CYP1A, belonging to the cytochrome P450-dependent monooxygenase enzyme subfamily, plays a significant role in the biotransformation of numerous xenobiotics. Its hepatic expression serves as a biomarker for detecting exposure to organic pollutants (Basilone et al., 2018; Passantino et al., 2014a). Here, the density of necrotic foci exhibited a higher occurrence in hatchery-produced males, in contrast to both hatchery-produced females and wild fish of both sexes. This heightened density of necrotic foci correlated with an increased presence of individual cells containing melanin, which likely corresponds to the monocyte/macrophage lineage. Additionally, this trend was associated with greater lipid accumulation within hepatocytes. In human biology, hepatic lipid accumulation can be influenced by multiple factors, including an elevated uptake of circulating fatty acids by the liver and reduced hepatic lipid export (Geisler and Renquist, 2017). In our study, hatchery-produced fish demonstrated a more pronounced accumulation of liver lipids compared to their wild counterparts. This discrepancy may point towards a potential hepatic distress of unknown origin in the cultured fish that could be attributed to the higher lipid content present in the diet provided to reared fish. Furthermore, the diminished lipid content observed in females might be attributed to heightened lipid consumption associated with the hepatic synthesis of vitellogenin (Castejón et al., 2021). Vitellogenin, a glycolipophosphoprotein with high density, is synthesized in the liver and subsequently transported *via* the bloodstream to the ovaries. There, it is internalized by oocytes during vitellogenesis, contributing to the formation of yolk proteins (Pousis et al., 2012, Pousis et al., 2018).

In conclusion, this study presents the initial findings on the presence of hepatic melanomacrophage centers in lumfish, a promising candidate for large-scale aquaculture production. These centers in lumpfish are characterized by small structures that exhibit similar constituents as observed in other fish species, including melanin, ferric ions within hemosiderin, and lipofuscin–ceroids. However, unlike some other fish species, melanomacrophage centers do not seem to serve as reliable indicators of lumpfish health status. This is evident from the lack of correlation between their density and numerical occurrence of necrotic foci or the lipid content within hepatocytes, both of which typically translate on hepatic distress.

## Funding information

This project has been generously funded by the H2020 ERANET BlueBio Cofund project, grant agreement 817992 (BESTBROOD). In addition, the 10.13039/501100005416Research Council of Norway, financially supported under project number 311799. JS has a PhD fellowship supported by Nord University.

## CRediT authorship contribution statement

**L. Passantino:** Conceptualization, Investigation, Writing – original draft, Visualization. **A. Corriero:** Conceptualization, Methodology, Resources, Writing – original draft, Writing – review & editing, Visualization, Project administration, Funding acquisition. **C. Pousis:** Investigation, Writing – review & editing. **R. Zupa:** Formal analysis, Investigation, Writing – review & editing, Visualization. **A. Perillo:** Investigation, Writing – review & editing. **J. Superio:** Investigation, Writing – review & editing. **J. Kumari Swain:** Investigation, Writing – review & editing. **A. Foss:** Investigation, Funding acquisition. **J. Galindo-Villegas:** Conceptualization, Writing – review & editing, Funding acquisition. **G. Ventriglia:** Investigation, Visualization, Writing – original draft.

## Declaration of Competing Interest

The authors declare that they have no known competing financial interests or personal relationships that could have appeared to influence the work reported in this paper.

## Data Availability

The data that support the findings of this study are available from the corresponding author upon request.
